# On Stereoscopic Art

**DOI:** 10.1177/20416695211007146

**Published:** 2021-05-27

**Authors:** Nicholas J. Wade

**Affiliations:** Psychology, University of Dundee, Dundee, United Kingdom

**Keywords:** anaglyphs, stereoscopic art, photography, graphics, random-dot stereograms, binocular vision, Wheatstone, Julesz, Dali

## Abstract

Pictorial art is typically viewed with two eyes, but it is not binocular in the sense that it requires two eyes to appreciate the art. Two-dimensional representational art works allude to depth that they do not contain, and a variety of stratagems is enlisted to convey the impression that surfaces on the picture plane are at different distances from the viewer. With the invention of the stereoscope by Wheatstone in the 1830s, it was possible to produce two pictures with defined horizontal disparities between them to create a novel impression of depth. Stereoscopy and photography were made public at about the same time and their marriage was soon cemented; most stereoscopic art is now photographic. Wheatstone sought to examine stereoscopic depth without monocular pictorial cues. He was unable to do this, but it was achieved a century later by Julesz with random-dot stereograms The early history of non-photographic stereoscopic art is described as well as reference to some contemporary works. Novel stereograms employing a wider variety of carrier patterns than random dots are presented as anaglyphs; they show modulations of pictorial surface depths as well as inclusions within a binocular picture.

Throughout the long history of pictorial art, attempts have been made to introduce the appearance of depth with marks on otherwise flat surfaces (see Brooks, 2017). The representation of pictorial depth was transformed by the announcement of the stereoscope (Wheatstone, 1838) and the depth that could be seen when two slightly dissimilar pictures are viewed, one to each eye. Moreover, Wheatstone’s appreciation of its impact on art was clearly stated: The depth seen with stereoscopic pairs was fundamentally different from that with a flat picture. As Ferragallo (1974) remarked: “I believe this paper describes one of the most remarkable techno-visual discoveries in the 35,000 years of the history of art” (p. 97). Wheatstone’s comments about art were made before the announcement of photography by Daguerre and Talbot in the following year although he was well aware of Talbot’s earlier experiments on capturing and fixing images with a camera (see Newhall, 1982; Schaaf, 1992; Wade, 1983).

Wheatstone also took photographs making what was probably the first “selfie” by a scientist in 1840 (Wade, 2014), and in the same year, he enlisted Talbot’s assistance to take stereoscopic photographs (Klooswijk, 1991). The first stereoscopic photographs were made by using a single camera and moving it laterally so that two slightly different photographs of the same scene were taken in succession. Various devices were introduced to avoid moving the whole camera, such as sliding bodies on a fixed base and moveable lenses (see Cox, 1978). This was to change with the introduction of twin-lensed (binocular) cameras that could take two slightly different photographs at the same time. Brewster announced his binocular camera as well as a description of his lenticular stereoscope in 1849; fuller accounts were presented 2 years later and in his book on the stereoscope (Brewster, 1849a, 1849b, 1851, 1856). Brewster used half-lenses in his stereoscope and binocular camera because he did not consider that it was then possible to grind two equal lenses. Dancer did just that with a twin-lensed binocular camera; he made his first model in 1852 and produced an improved, commercially available model in 1856 (see Dancer, 1886, for a description of his early binocular cameras). Binocular cameras, such as those of Brewster and Dancer, removed many of the difficulties of alignment and time difference associated with using a single camera for stereoscopic photographs. When combined with Brewster’s stereoscope, they did much to hasten the popularity of stereoscopic photography in the second half of the 19th century. The situation was summarised by Brewster:

The photographic camera is the only means by which living persons and statues can be represented by means of two plane pictures to be combined by means of the stereoscope; and but for the art of photography, this instrument would have had a very limited application. (1856, p. 135)

## Stereoscopic Viewing

The oldest method for appreciating different views of an object with each eye is to close them in turn and compare what is seen (Wade, 1998). Wheatstone (1838) described earlier methods of viewing dissimilar pictures with two eyes either by over- or under-convergence; this was often assisted by simple viewing devices (Wade, 1987; Wade & Ngo, 2013; Wade & Ono, 2012). The first stereoscopes were based on mirrors, prisms, or lenses (Brewster, 1849b; Wheatstone, 1838, 1852), but other systems for separating the images presented to each eye were enlisted. After Wheatstone invented both reflecting (mirror) and refracting (prism) stereoscopes in the early 1830s, some simple and novel optical techniques were introduced. Many of these involving mirrors and prisms were illustrated by Brewster (1851) as well as by Dove (1851). The most popular model of stereoscope was Brewster’s lenticular version. The optical manipulation of disparities was also achieved with Wheatstone’s (1852) pseudoscope, which reversed them, and with Helmholtz’s (1857) telestereoscope, which exaggerated them. Cross-polarised projections and viewing glasses were devised by Anderton between 1891 and 1895; he mounted two Nicol prisms in the paths of light from two projectors and viewed the images through two Nicol prisms in opposite orientations mounted in viewing glasses; stereoscopic images were projected onto a specially silvered screen (see Anderton, 1895). Anderton appreciated that this provided a method for presenting and viewing stereoscopic images, and the system became popular at the end of the 19th century (McBurney, 2020). When sheet polarisers were manufactured in the 1930s, the technique became more widely used. The advantage of this technique is that coloured images can be seen in depth or rivalry. Examples of the vast range of 19th century stereoscopes are illustrated in Wing (1996), and descriptions of more recent stereoscopic techniques can be found in Blundell (2011) and Howard and Rogers (1995).

**Figure 1.**
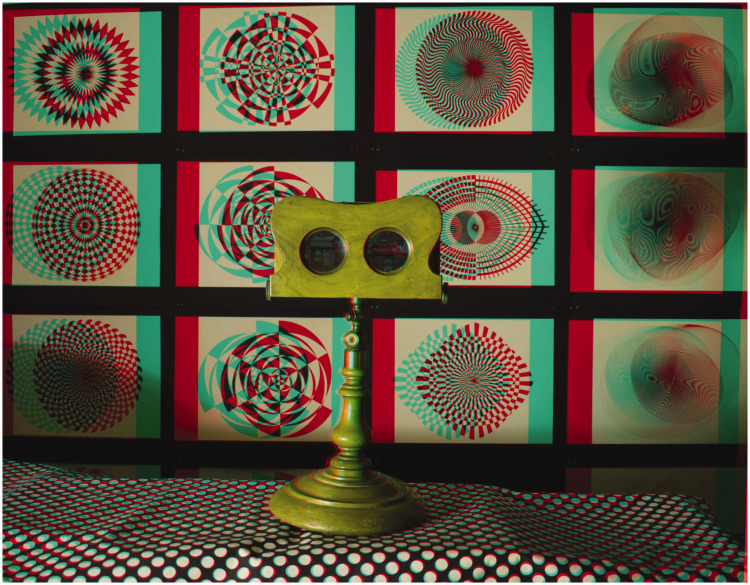
*Stereoscopic stereoscope* by Nicholas Wade.

Anaglyphs are displays in which the left and right eye images are printed in different colours, such as red and cyan, and they are viewed through filters of the same colours. They have typically been used to present slightly different images to each eye so that they are seen in stereoscopic depth. The use of colours for separating the eyes to see depth was realised by Rollmann (1853); the colours that he found worked best were blue and yellow drawings combined with red and blue glasses. D’Almeida (1858) described a similar system using images projected with two magic lanterns having colour filters in front of the lenses; the observer viewed the superimposed projections through similar filters, one for each eye. He found that combinations of red and green projections and glasses worked well. Ducos du Hauron devised a method of over-printing red and blue or green designs in 1891 (see Ducos du Hauron, 1897), and it was referred to as the art of the anaglyph (see Wade, 2021). Thereafter, anaglyphs became increasingly popular as a means for printing stereoscopic drawings and photographs. The general standard now is for red/left eye, cyan/right eye filters for viewing similarly coloured printed images and these are recommended for viewing the anaglyphs in this article. Figure 1 presents an anaglyphic photograph of a Brewster-type pedestal stereoscope which appears in depth when viewed with the red/left eye, cyan/right eye arrangement. However, reversing the filters and therefore the signs of the disparities does not reverse the apparent depth: monocular cues of occlusion override those for disparity.

## Stereoscopic Art

The term *stereoscopic art* is assigned to those pictorial works that are dependent on the operation of two eyes in cooperation to yield an impression of depth that is not available to either eye alone. There has been much sterile debate about whether photography is pictorial art, and there is little virtue in adding to it. However, the debate is essentially pointless in the context of stereoscopic art as it did not exist prior to the almost synchronous inventions of stereoscopy and photography.

Stereoscopes provided not only precise instruments for investigating the science of vision with two eyes, but they also opened up a new world of art. In his first memoir, Wheatstone appreciated the challenges stereoscopic drawing and painting presented to artists:

It will now be obvious why it is impossible for the artist to give a faithful representation of any near solid object, that is, to produce a painting which shall not be distinguished in the mind from the object itself. When the painting and the object are seen with both eyes, in the case of the painting two *similar* pictures are projected on the retinae, in the case of the solid object the pictures are *dissimilar*. (1838, p. 372)

In his second memoir, he astutely observed “What the hand of the artist was unable to accomplish, the chemical action of light, directed by the camera, has enabled us to effect” (1852, p. 7). Wheatstone’s statement can be amplified when the computer is added to the camera. Indeed, it was with computer-generated random-dot patterns that Julesz (1960) was able to realise Wheatstone’s dream of generating stereoscopic depth without presenting clues to depth in the monocular components.

The stereoscopic figures displayed by Wheatstone (1838) were almost all simple line drawings:

For the purposes of illustration I have employed only outline figures; for had either shading or colouring been introduced it might be supposed that the effect was wholly or in part due to these circumstances, whereas by leaving them out of consideration no room is left to doubt that the entire effect of relief is owing to the simultaneous perception of the two monocular projections, one on each retina. (p. 376)

**Figure 2.**
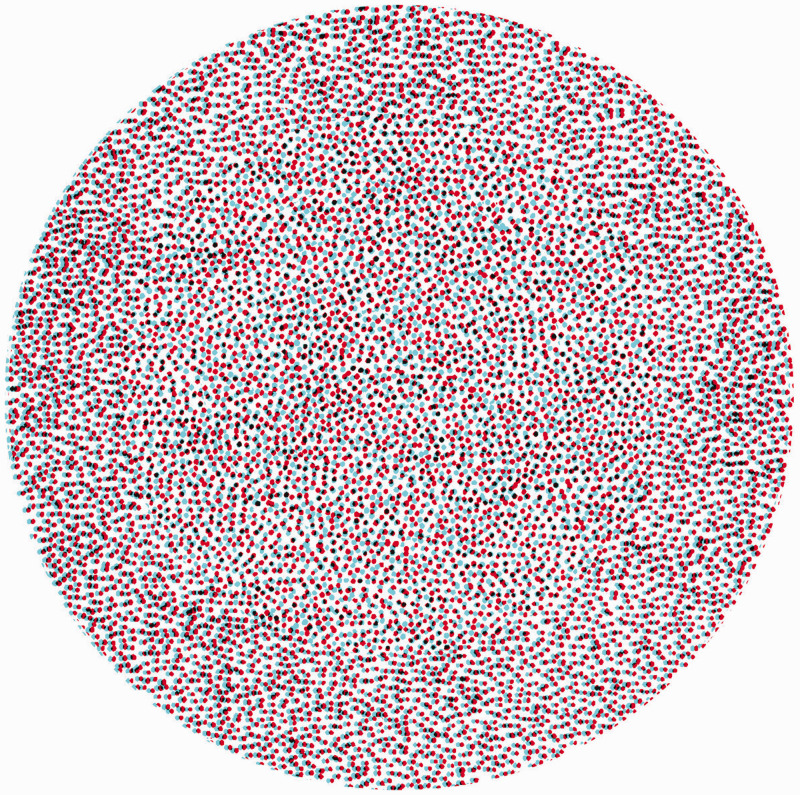
*Dots in depth* by Nicholas Wade.

That is, he wished to avoid the usual painterly techniques for conveying depth or distance so that only disparity was operating. He could not exclude cues to depth in the monocular views, but he could reduce them. Wheatstone could not fulfil his desire, but it was achieved over a century later by Béla Julesz (1960, 1971, 1978; see Papathomas et al., 2019) with his random-dot stereograms; they enabled stereoscopic depth perception to be investigated independently of monocular object recognition. Those devised by Julesz are pairs of matrices of squares in which the contents of each cell are randomly assigned as black or white; displacing a region in one display and combining them in a stereoscope results in that region appearing in depth. An anaglyphic example is shown in Figure 2 which is comprised of hand-drawn dots rather than computer-generated patterns. Each component pattern of dots appears flat when viewed with either eye alone but depth emerges when both eyes are used: A small central circle is seen relative to six annuli at different depth planes. With the red/left eye and cyan/right eye, the annuli appear to recede, whereas they approach with a red/right and cyan/left combination. It can take some seconds for the depths to articulate.

Julesz was not the first to make abstract stereoscopic patterns. The use of random dots to conceal patterns monocularly and reveal them stereoscopically was devised by the microanatomist Ramón y Cajal as early as 1870, as he later described:

During my stereoscopic honeymoon, that is to say, long ago between the years 70 and 72, I was absorbed in imagining new fancies and recreations of this genre. My aim was to achieve a mysterious writing, which could only be deciphered with the stereoscope and usable for those people who don’t want to divulge their own matters … . The game consists of making a proof [a print on glass] only with dots, lines and scribbles, or also of letters, crossed and entangled in a thousand ways. A proof in which, with the naked eye, you cannot read anything at all. And, nevertheless, as soon as you see the double image of this background in the stereoscope, a perfect legible sentence or text suddenly appears, standing out on the foreground and clearly detaching itself from the chaos of the lines or dots. (Ramón y Cajal, 1901, translated in Bergua & Skrandies, 2000, p. 71)

The separated patterns on glass and board were photographed with a binocular camera, and the two half-images were viewed in a stereoscope. It seems likely that Herbert Mobbs (Secretary to the Stereoscopic Society for 40 years) used a similar technique in 1919 for making two random-dot patterns in which the letter L was visible in depth when combined binocularly by over- or under-convergence. Dots of different sizes were drawn on one surface and then reproduced on a transparent surface placed over it, adding dots in the shape of the letter; the two surfaces were then photographed separately and mounted side by side. It had the amusing title “Simple Monoscopist: ‘Ah! The Heavens’. Sophisticated Stereoscopist: ‘No! L.’” (Dennis & Patterson, 1995, p. 26, where the stereogram is illustrated). Paired patterns of dots were produced by Kompaneysky (1939; see Blundell, 2011; Howard & Rogers, 2002) and by Aschenbrenner (1954; see Shipley, 1971) which carried a face or a word, respectively, when combined in a stereoscope; illustrations of both stereograms can be found in Blundell (2011) and Howard and Rogers (2002). Aschenbrenner was stimulated by his work on revealing camouflaged objects from aerial stereoscopic photographs and he devised a novel means of simulating this:

The image was designed to be analogous to the random distribution of terrain features and shapes in an aerial photograph. The process used is an extremely simple one and can be briefly described as follows: A large quantity of black and white discards from a paper punch were thoroughly mixed together to assure an even mixture of distribution. The mixture was strewn on a large surface so that the density completely covered the surface. A single photograph was taken of the surface which is the background image of the stereogram. One set of letters was cut from a copy of the image, superimposed onto the background image, and photographically recorded. The letters were then displaced laterally by 1½ mm on the background photographic image and a second photographic record was produced. (The original photographs are 9 cm by 12 cm and are separated by 24 cm). (Shipley, 1971, p. 1491)

Revealing otherwise hidden objects in aerial stereoscopic photographs was also the stimulus for Julesz to make random-dot stereograms, but he was able to harness the power of computers in creating them: “After all, to break camouflage in aerial reconnaissance, one would view aerial images taken from two different positions (the use of parallax) through a stereoscope, and the camouflaged target would jump out in vivid depth” (Julesz, 1991, p. 745). What distinguished his experiments was not only the use of computer-generated patterns but also an appreciation of their significance in furthering understanding of stereoscopic vision:

The investigation reported here utilized patterns devoid of all cues except binocular parallax, by using artificially created stereo images with known topological properties. Such visual displays ordinarily never occur in real-life situations, and a digital computer (with a video transducer at its output) was programmed to generate them. When these unfamiliar pictures are viewed stereoscopically, peculiar and often unexpected depth effects can be seen. In addition, the perception time of depth under such circumstances is sometimes in the order of minutes (instead of the few milliseconds required for familiar stereo images). This slowing down of the visual process facilitated the present investigation without having much effect on the stability of depth impression after depth was finally perceived. (Julesz, 1960, p. 1126)

Julesz recognised the artistic possibilities presented by random-dot stereograms. Indeed, together with Michael Noll, he instigated one of the first exhibitions of computer-generated art in 1965 (see Noll, 2016). He also interacted with Salvador Dali over several years with regard to Dali’s stereoscopic works (Julesz, 1995; Tyler, 2014a; Weibel, 2004). Most notably, Dali made a random-dot anaglyphic painting as homage to Julesz (see http://art-dali.com/1970_67.html) . Julesz reminisced:

To my further amazement, there were “artists” who used several of my images in collages. When Salvador Dali invited me to his studio and showed me his recent work, I was honored. There was a picture of Christ nailed on a cross made of the DNA double helix. Below his feet, two silk scarves cascaded downward, creating a moiré effect, and around the figure’s head were a stereo viewer and a cutout of an RDS depicting a torus, taken from my Scientific American article (Julesz, 1965). This torus had been melted and served as a halo for the crucified Jesus. I knew immediately that I had finally “made it.” After this first encounter with Dali, he asked my advice several times, particularly when he was painting some large stereo pairs at an angle with a half-silvered mirror between them. He also asked me to supply him with some random-dot stereograms, so that he could modify them according to his taste. I mention this episode merely to illustrate that the impact of the RDS even permeated art. Because I was impressed by Dali’s knowledge of perception during our conversations, I feel that he paid tribute to the RDS not just as an artist, but also as a colleague well versed in my specialty. (Julesz, 1995, reprinted in Weibel, 2004, p. 123)

Most of Dali’s paired paintings for stereoscopic viewing are relatively small, but he did paint some large ones. For example, the dimensions of *La chaise. Œuvre stéréoscopique* (https://www.salvador-dali.org/fr/oeuvre/catalogue-raisonne-peinture/obra/882/la-chaise-oeuvre-stereoscopique) from 1975 were 4 metres high by 2 metres wide, and it was made to be viewed with large mirrors, essentially as a Wheatstone mirror stereoscope (see King, 2018). Like Dali, Marcel Duchamp followed developments in vision science and often incorporated new phenomena in his art works (see Ades, 2000, 2008). This also applied to stereoscopy, and he produced a range of binocular works from 1918 onwards. Some involved additions by hand to existing stereoscopic photographs and others were anaglyphs (King, 2018). Both Dali and Duchamp made works in which the left and right eye images were radically different and engaged in binocular rivalry. In the 1970s, Alfons Schilling produced hand-drawn random-dot stereograms and autostereograms as well as constructing elaborate pseudoscopes in his explorations of binocular vision (Schilling, 1997, 2004). The constructionist artist Terry Pope has made pseudoscopes and hyperscopes that are based on reflection rather than refraction together with constructions that are to be viewed with them (see http://terry-pope.com/). Calum Colvin has returned to the pioneers of stereoscopy to capture them in depth as well as presenting the paired pictures in a range of viewing devices. Not only are Wheatstone and Brewster shown in stereoscopic depth but also in binocular rivalry and the Chimenti figures are also displayed (see Colvin, 2009; Wade, 2009). Depth derived from disparity vies with pictorial depth so that the works are not narrowly stereoscopic, but they display a dynamic duel between the pictorial and binocular cues to depth.

Dali did introduce an element of obfuscation to the history of stereoscopic art by suggesting that the 17th century Dutch artist, Gerrit Dou, painted stereoscopic works. While this has been questioned on art historical grounds (Wallis, 2015), similar claims for works by Jacopo Chimenti and Leonardo da Vinci have been rejected on experimental grounds. The controversy over whether two drawings from around 1600 by Chimenti were stereoscopic stirred the world of science in the 19th century (see Wade, 2003); the doubt placed on the proposal at the time has been supported by psychophysical experiments by Brooks (2017). Brooks has also found little evidence to support suggestions by Carbon and Hesslinger (2013) that two versions of Leonardo’s *Mona Lisa* were intended for stereoscopic viewing.

As Wheatstone (1852) predicted, there was a surge in stereoscopic photography which produced many fine examples of the genre (see Pellerin & May, 2014; Taylor, 2018). The demand for stereoscopic photographs was supplied by an increasing number of manufacturers in Europe and America, such as Negretti & Zambra (founded in 1850), The London Stereoscopic Company (1856), Underwood & Underwood (1881), and the Keystone View Company (1892). They also marketed stereoscopes, and Figure 1 shows a model (Scott’s Patent Stereoscope) from 1856 sold by Negretti & Zambra. Relatively few graphic artists ventured into this new world to compete with the camera but some did. These were mostly concerned with the science or clinical testing of stereoscopic vision, producing graphic designs of outline geometrical figures. In the 20th century, abstract stereoscopic paintings and graphics were made by Fischinger (see Zone, 2006) and Ferragallo (1974) prior to the widespread use of computers towards the end of the century. Wilding (1977, 2007; see Kondo et al, 1990; Wade, 2007) has produced anaglyphic art as well as geometrical and abstract works by creating depth with superimposed and separated gratings. Disparities were between the relative locations of moiré fringes in each eye, and the stereoscopic depth changed with the movements of the observer towards or away from the works. Ninio (1994, 2001, 2011) has produced computer-generated patterns of stunning symmetry which are seen in relative depth when viewed stereoscopically.

**Figure 3.**
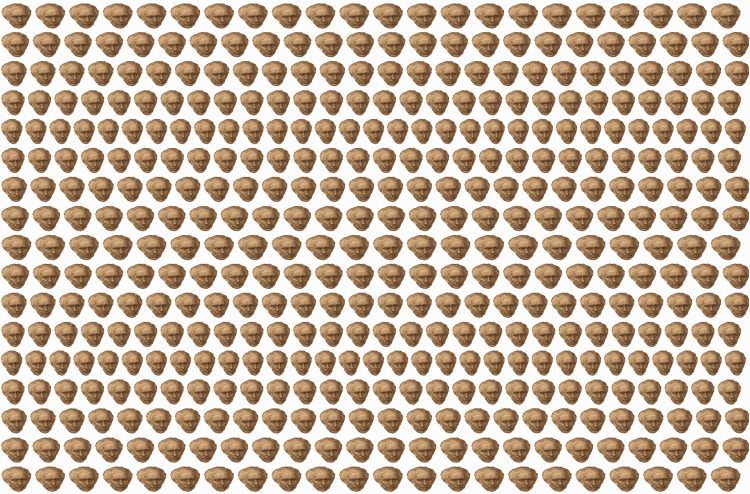
*Autostereoscopic portraits of Brewster* by Nicholas Wade. An array of portraits of Brewster with rows varying systematically in size and separation. Combining adjacent pairs by over- or under-convergence will lead to the appearance of parallel, horizontal humps and hollows.

Presenting regular and repetitive dot patterns that enable fusion of neighbouring pairs provides the basis of the wallpaper illusion. This binocular depth effect was initially described by Blagden (1813) not in wallpaper but in the fluted marble of a chimney piece. With under-convergence so that adjacent elements were fused the fluting appeared to be further away and magnified relative to fixating on the same elements. However, its significance was not appreciated until after the invention of the stereoscope when Brewster (1844) rediscovered the illusion when observing a repetitive pattern of flowers printed on wallpaper. It was from such patterns that were frequently printed on wallpaper that the phenomenon derived its name. When equivalent but laterally separated patterns are combined binocularly, they seem suspended in the plane of convergence. If slight variations in the locations of the repetitions along rows are introduced, then more complex depth planes are visible and aspects of disparity processing become involved, as in Figure 3. This is the principle employed in autostereograms (Tyler, 2014b; Tyler & Clarke, 1990). Wallpaper illusions and autostereograms can be seen without the aid of any viewing device; they involve dissociating convergence from accommodation by converging the eyes to combine neighbouring elements or viewing them with parallel visual axes.

Attempts to combine stereoscopic vision with apparent motion were considered by Wheatstone and others (Wade, 2012). Indeed, Pellerin (2017) has suggested that the first movies were made for the stereoscope. The rich history of cinematic stereo has been chronicled by Zone (2007) and others but will not be pursued further here.

## Anaglyphic Art

Julesz’s achievement in advancing stereoscopic art is celebrated here with a wider variety of textures (carrier patterns) than random dots for disparities, and they are displayed as anaglyphs. The carrier patterns are not only more complex, but they can also have an appeal independently of the depth they contain. The starting points for the illustrations were either graphic designs or natural textures. They were scanned or photographed and digitally modified to produce the carrier patterns that could be paired and combined to make the anaglyphs with StereoPhoto Maker software (http://stereo.jpn.org/eng/stphmkr/).

The illustrations that follow are examples of what can be called anaglyphic art, an example of which is shown in Figure 4. When viewed without red/cyan glasses, the symmetrical design has a complex structure that is derived from a photograph of leaves on a forsythia bush. With the red filter in front of the left eye and the cyan filter before the right eye then a word is seen beyond the plane of the background texture; reversing the filters reverses the depth.

**Figure:**
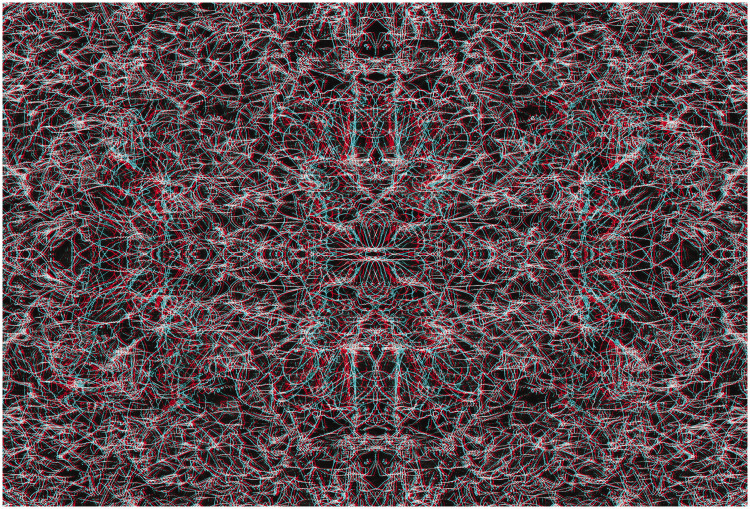
Figure 4. *Stereoscopic ART* by Nicholas Wade.

**Figure 5.**
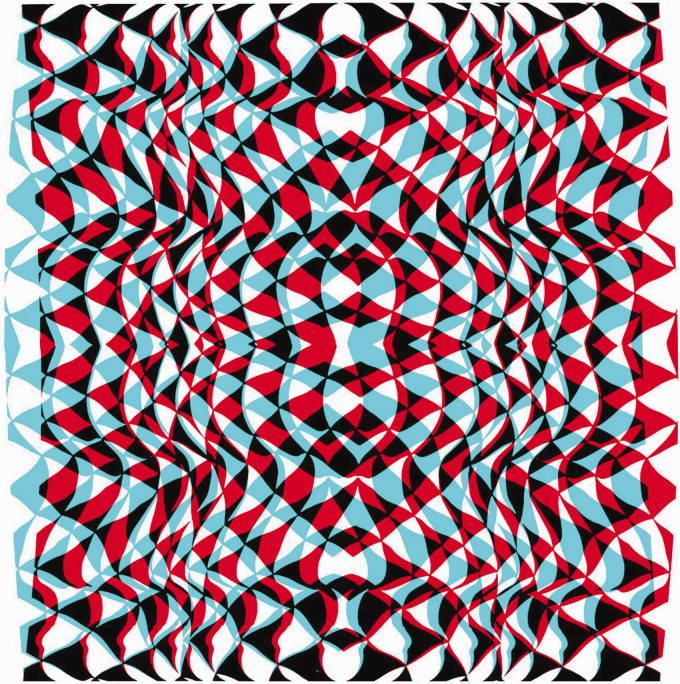
*Curvaceous borders* by Nicholas Wade.

The carrier patterns can be graphic designs, photographs of natural textures, or manipulated photographs. Stereoscopic depth can be induced by disparities over the whole pattern surface or of contents within it or both. Most non-stereoscopic graphic designs are two dimensional; they are produced to display the interactions of contours and colours. Variations in structure between two designs can introduce depth in ways that are difficult, if not impossible, to achieve with photographs of objects. The left and right eye images can readily be seen by closing each eye in turn so that the red/cyan separations are visible. Often the stereoscopic effect is not visible initially and so some patience might be required for the depth to emerge. Unlike conventional stereoscopic photographs, reversing the filters will reverse the depth seen. Figure 5 is based on a graphic design the surface of which appears to be concave or convex depending upon the arrangement of the filters in front of the eyes.

As noted earlier, there is relatively little abstract stereoscopic painting, but it is possible to introduce stereoscopic depth to abstract works, as in Figure 6, which contains several depth planes. Not only does the pictorial surface appear in twisted depth but the discs in each quadrant are also at different stereoscopic depths.

**Figure:**
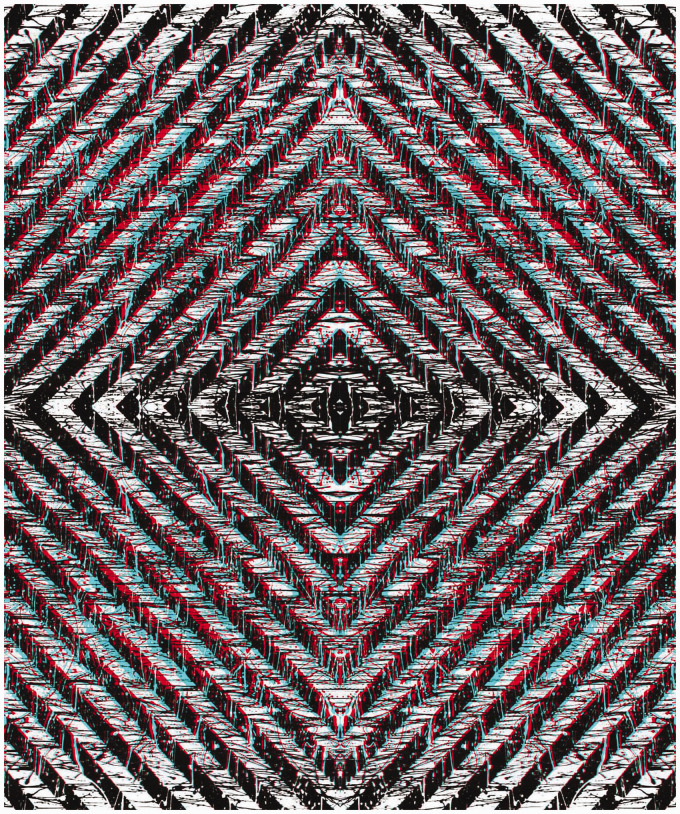
Figure 6. *Twisted* by Nicholas Wade.

**Figure 7.**
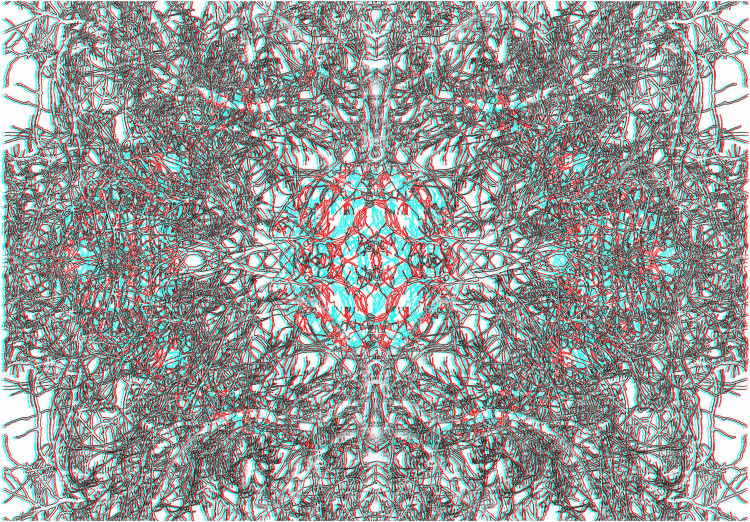
*Window on the world* by Nicholas Wade.

Figure 7 contains a schematic eye within the carrier pattern that is derived from a photograph of branches and twigs. Branches are an appropriate motif as the visibility of the retinal blood vessels is called the Purkinje tree (Purkinje, 1823; see Wade & Brožek, 2001).

**Figure 8.**
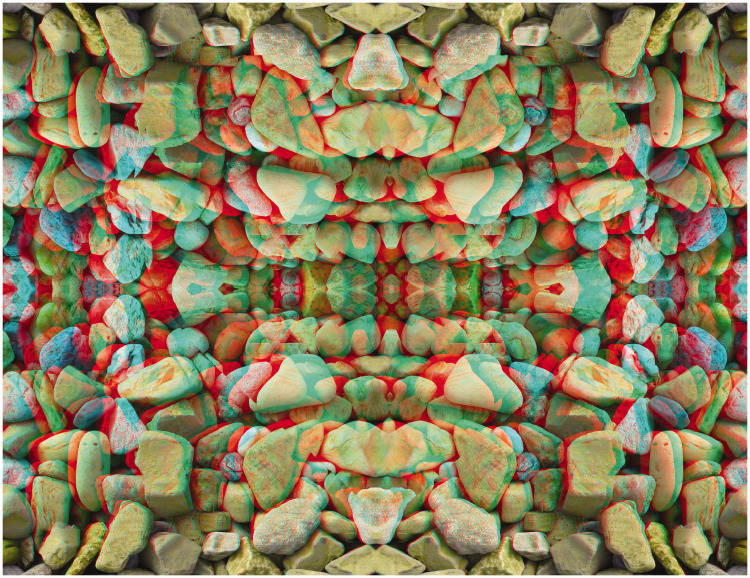
*Heart of stone* by Nicholas Wade.

Many manipulations can be made with stereoscopic designs both in terms of the carrier configuration as well as the form of the apparent depth within it. The apparent form of a pictorial surface can be modulated as can the relative depths of different parts. Indeed, both of these can be varied together as is shown in Figure 8: The depth in the surface is echoed in the shape that is in depth with respect to it. The carrier pattern is derived from a photograph of stones on a beach.

**Figure 9.**
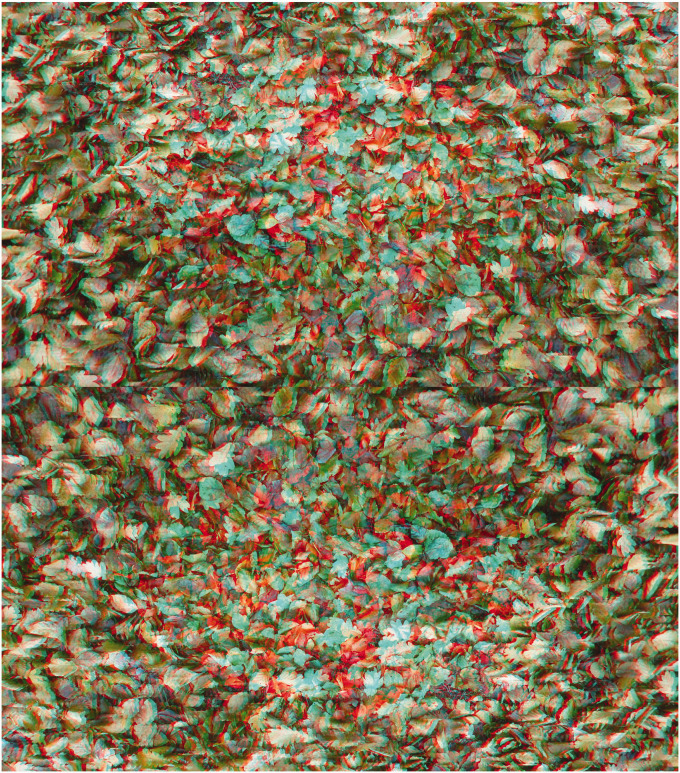
*Leaf fall* by Nicholas Wade.

The elements in depth can mirror features of the pattern carrying the disparities, as in Figure 9 in which leaves can be seen rising or falling with respect to the bed of autumnal leaves.

**Figure 10.**
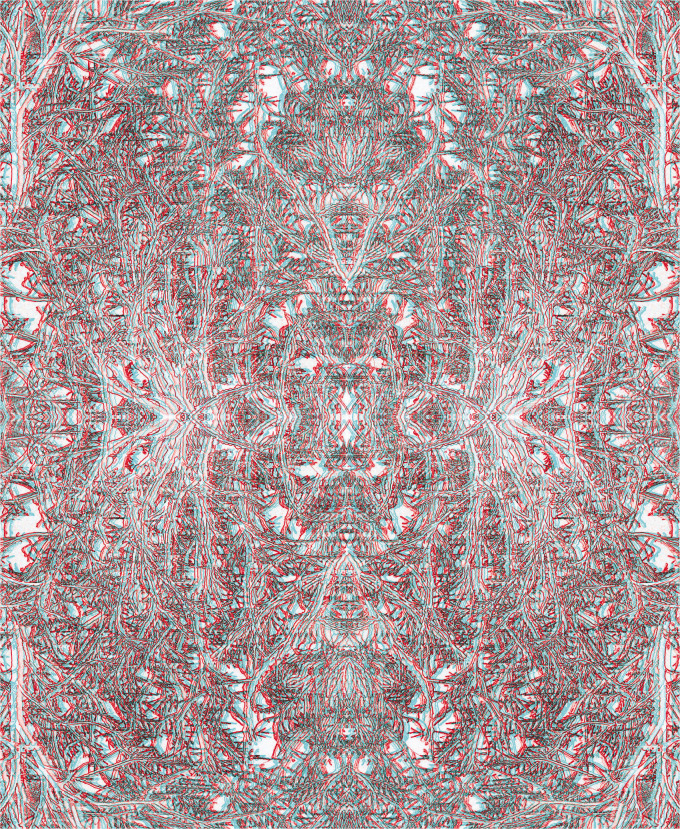
*Stereoscopic ambiguity* by Nicholas Wade.

Julesz (1971) also manipulated the structure of the regions in depth to create complex figures like random-dot versions of the Poggendorff and Müller-Lyer illusions. Figure 10 carries a perceptual puzzle. The carrier design is derived from a photograph of snow-covered branches on a chestnut tree. The shape at the centre of the design is a well-known figure/ground ambiguity. When the components are defined by stereoscopic depth, the ambiguity is attenuated, as can be seen by reversing the red/cyan glasses; the apparently nearer part is seen as the figure (see Tyler & Kontsevich, 1995).

**Figure 11.**
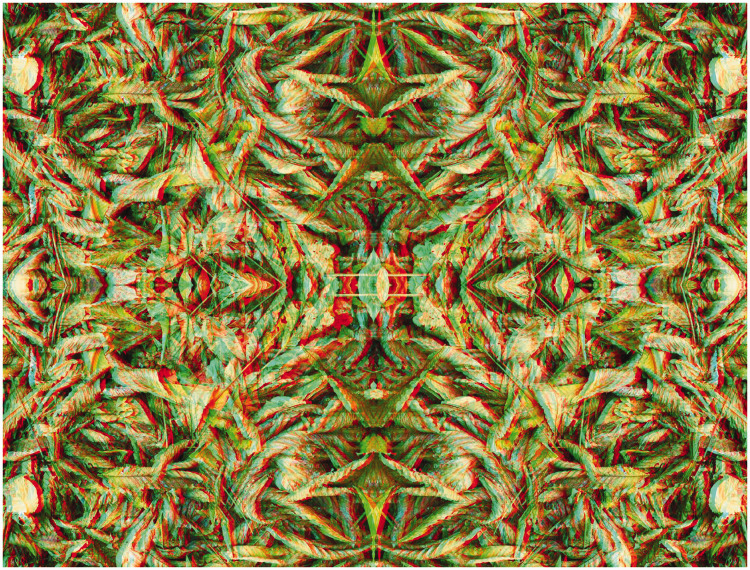
*These are not tobacco leaves (Homage to Magritte)* by Nicholas Wade.

Many artists have mused on the relationship between their representations of objects and the objects themselves. Prominent among these has been René Magritte who drew attention to this in a series of pipe paintings in which beneath a depicted pipe were the painted words (in French) “This is not a pipe” (see Foucault, 1982). Magritte was stating that both written words and images are different representations of objects, one of the category pipes and the other of identity of the particular pipe shown (see Wade, 1990). The theme is echoed in Figure 11; patterns made up from tobacco leaves initially conceal a picture of a curved briar pipe within them which cannot be seen with either eye alone but emerges stereoscopically.

## Conclusion

The desire to produce stereoscopic pictures devoid of monocular depth cues was voiced by Wheatstone but was not realised until Julesz produced random-dot stereograms. The momentum generated by Julesz, particularly by his book *Foundations of cyclopean perception*, continues to stimulate scientists and artists to seek novel graphical as well as computer graphical techniques for broadening the scope of stereoscopic art.
